# The Role of Atypical Cannabinoid Ligands O-1602 and O-1918 on Skeletal Muscle Homeostasis with a Focus on Obesity

**DOI:** 10.3390/ijms21165922

**Published:** 2020-08-18

**Authors:** Anna C. Simcocks, Lannie O’Keefe, Kayte A. Jenkin, Lauren M. Cornall, Esther Grinfeld, Michael L. Mathai, Deanne H. Hryciw, Andrew J. McAinch

**Affiliations:** 1 Institute for Health and Sport, Victoria University, Melbourne, VIC 8001, Australia; anna.roy@live.vu.edu.au (A.C.S.); Lannie.OKeefe@vu.edu.au (L.O.); K.Jenkin@westernsydney.edu.au (K.A.J.); laurenc@outlook.com.au (L.M.C.); estherggrinfeld@gmail.com (E.G.); michael.mathai@vu.edu.au (M.L.M.); d.skelly@griffith.edu.au (D.H.H.); 2 First Year College, Victoria University, Melbourne, VIC 8001, Australia; 3 School of Science, Western Sydney University, Campbelltown, NSW 2560, Australia; 4 The Florey Institute of Neuroscience and Mental Health, Melbourne VIC 3052, Australia; 5 School of Environment and Sciences, Griffith University, Nathan, QLD 4111, Australia; 6 Environmental Futures Research Institute, Griffith University, Nathan, QLD 4111, Australia; 7 Australian Institute for Musculoskeletal Science (AIMSS), College of Health and Biomedicine, Victoria University, Melbourne, VIC 8001, Australia

**Keywords:** Atypical Cannabinoids, O-1602, O-1918, GPR18, obesity and skeletal muscle

## Abstract

O-1602 and O-1918 are atypical cannabinoid ligands for GPR55 and GPR18, which may be novel pharmaceuticals for the treatment of obesity by targeting energy homeostasis regulation in skeletal muscle. This study aimed to determine the effect of O-1602 or O-1918 on markers of oxidative capacity and fatty acid metabolism in the skeletal muscle. Diet-induced obese (DIO) male Sprague Dawley rats were administered a daily intraperitoneal injection of O-1602, O-1918 or vehicle for 6 weeks. C_2_C_12_ myotubes were treated with O-1602 or O-1918 and human primary myotubes were treated with O-1918. GPR18 mRNA was expressed in the skeletal muscle of DIO rats and was up-regulated in red gastrocnemius when compared with white gastrocnemius. O-1602 had no effect on mRNA expression on selected markers for oxidative capacity, fatty acid metabolism or adiponectin signalling in gastrocnemius from DIO rats or in C_2_C_12_ myotubes, while APPL2 mRNA was up-regulated in white gastrocnemius in DIO rats treated with O-1918. In C_2_C_12_ myotubes treated with O-1918, PGC1α, NFATc1 and PDK4 mRNA were up-regulated. There were no effects of O-1918 on mRNA expression in human primary myotubes derived from obese and obese T2DM individuals. In conclusion, O-1602 does not alter mRNA expression of key pathways important for skeletal muscle energy homeostasis in obesity. In contrast, O-1918 appears to alter markers of oxidative capacity and fatty acid metabolism in C_2_C_12_ myotubes only. GPR18 is expressed in DIO rat skeletal muscle and future work could focus on selectively modulating GPR18 in a tissue-specific manner, which may be beneficial for obesity-targeted therapies.

## 1. Introduction

Obesity rates and associated co-morbidities such as type two diabetes mellitus (T2DM) are increasing world-wide [1]. Pharmaceutically targeting these conditions may be beneficial in combination with a healthy diet and increased physical activity to help reduce the health-related costs and burdens for an individual, community and government.

Skeletal muscle is an important regulator of whole body energy expenditure and is a major determinant for resting energy expenditure in humans [2]. The skeletal muscle is a major site for glucose and fatty acid oxidation as well as insulin action and is an organ that is highly adaptable to environmental stressors such as obesity [3]. In obesity, there is an increased triglyceride content within skeletal muscle [4], which is associated with insulin resistance [5]. Pharmacologically modulating the skeletal muscle to alter signalling pathways and improve metabolic homeostasis in obesity and associated co-morbidities such as T2DM may therefore be beneficial. The skeletal muscle is heterogeneous in nature and is composed of different fibre phenotypes [6]; these fibre phenotypes can have either a more oxidative or glycolytic characteristic. The endocannabinoid system has previously been a pharmacological target for obesity [7]. As such, peripheral modulation of this system, particularly in the skeletal muscle, may be beneficial. Cannabinoid receptors (CB) CB_1_ and CB_2_ are expressed in the skeletal muscle [8], with CB_1_ influencing oxidative pathways [9]. Limited research has focused on atypical cannabinoid compounds and receptors within the skeletal muscle in obesity.

O-1602 and O-1918 are synthetic derivatives of cannabinoid compounds, which have an affinity/putative affinity to the putative cannabinoid receptors G Protein-Coupled Receptor (GPCR) 55 (GPR55) and GPR18 [10,11,12,13,14]. The putative cannabinoid receptor GPR55 [10] appears to have a role in regulating energy homeostasis [15]; GPR55 expression is up-regulated in the adipose tissue of obese humans when compared with non obese humans [16]. GPR55 deficiency in mice is associated with increased adiposity, reduced physical activity and energy expenditure as well as impaired insulin signalling in peripheral metabolic tissues [17]. GPR55 knockout mice have a slightly increased fasting plasma insulin compared with wild type mice, albeit not statistically significant [17]. GPR55 is expressed in the skeletal muscle obtained from a range of different species and cell lines including wild type mice (gastrocnemius), rat (soleus), L6 myotubes and human primary myotubes [17]; additionally, GPR55 expression appears to be increased when L6 cells are differentiated, as well as with pre-treatment with an endogenous ligand of GPR55, lysophosphatidylinositol (LPI) [17]. GPR18 expression has recently been verified in vascular smooth muscle obtained from human placenta [18] and previously has been shown to be expressed in cardiac tissue obtained from diabetic rats [19]. Currently, the expression of GPR18 in the skeletal muscle and thus its role, if any, in obesity is unknown.

Activation of GPR55 and GPR18 by O-1602 has previously been shown to enhance intracellular calcium mobilisation [13,20]. Recently, O-1602 enhanced intracellular calcium transients in mouse insulinoma (MIN6) pancreatic mouse β cell line, which led to an increase in insulin secretion; this study also showed that GPR55 protein was expressed in the MIN6 cell line [21]. GPR18 signalling, however, is complex and it has been found that either atypical cannabinoid ligand O-1602 or O-1918 enhance calcium-mediated mobilisation and MAPK activity, but the ligands did not influence β-arrestin translocation [13]. O-1918 has been suggested as a biased ligand for GPR18 with agonist activity [13], while others have found O-1918 to act as an antagonist for GPR18 [11]. In the skeletal muscle, increases in intracellular calcium are associated with muscle contraction positively influencing skeletal muscle homeostasis. Pharmacological modulation of GPR55 (unless otherwise stated) and GPR18 in the skeletal muscle could alter markers such as; nuclear factor of activated t-cells (NFAT), peroxisome proliferator-activated receptor-gamma coactivator alpha (PGC1α), pyruvate dehydrogenase kinase 4 (PDK4), adaptor protein, phosphotyrosine interaction, PH domain and leucine zipper containing 1 (APPL1), which are influenced by calcium signalling [22,23,24,25] and may be a beneficial treatment for obesity.

Previous research indicates that atypical cannabinoid O-1602 has a role in energy homeostasis by increasing adiposity in rats [26]. The effect that O-1918 has on energy homeostasis is currently unclear, although cannabidiol (CBD), an analogue of O-1918, promotes a browning phenotype and lipolysis, while reducing thermogenesis and lipogenesis in 3T3-L1 adipocytes [27]. Abnormal cannabidiol (Abn-CBD), an analogue of O-1602, enhanced GPR18 expression in cardiac tissue obtained from diabetic rats; Abn-CBD restored both circulating and cardiac concentrations of adiponectin and nitric oxide, and diminished oxidative stress in diabetic rats, while O-1918 blunted these observed favourable effects in this model [28]. Our group has previously shown that chronic administration of atypical cannabinoid compounds do have an effect on whole body energy homeostasis in a rodent diet-induced obesity (DIO) model [29]. Specifically, in this DIO model, O-1602 reduced bodyweight, body fat and improved albuminuria, although it had adverse effects on the liver and kidney [29]. In the DIO model, O-1918 improved albuminuria, in the absence of an effect on body weight or total body composition [29]. Further, O-1918 treatment up-regulated a number of circulating pro-inflammatory cytokines and reduced the mass of brown fat pads, while having no effect on white fat pad mass [29]. While the atypical cannabinoids O-1602 and O-1918 appear, overall, not to have desirable effects in DIO systemically, understanding the effects that these compounds have on organs involved in the regulation of energy homeostasis, such as the skeletal muscle, may be beneficial.

This study aimed to determine whether GPR18 is expressed in the skeletal muscle of DIO rats and whether there is a variation in expression of GPR18 between red or white gastrocnemius skeletal muscle in either the presence or absence of O-1602 and O-1918. This study further aimed to determine the effect abnormal cannabinoid compounds O-1602 and/ or O-1918 have on the expression of markers involved in skeletal muscle homeostasis in a number of models including; C_2_C_12_ myotubes, whole muscle obtained from DIO rats, and human primary myotubes derived from obese and T2DM individuals.

## 2. Results

### 2.1. GPR18 Expression in Red and White Gastrocnemius in the Absence and Presence of Atypical Cannabinoids in DIO

**Figure 1:**
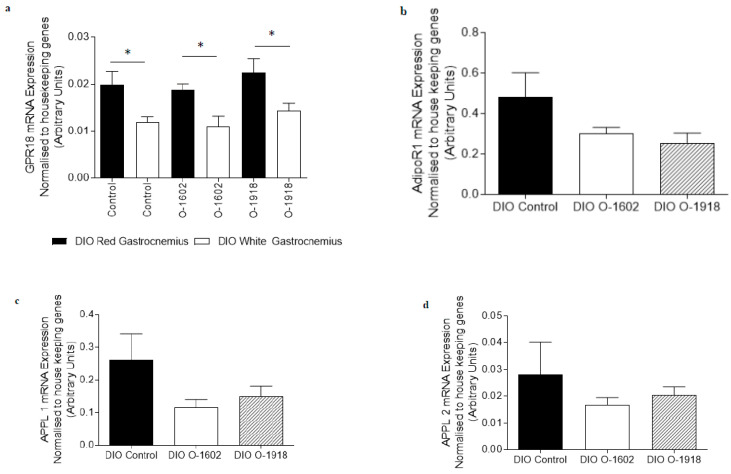

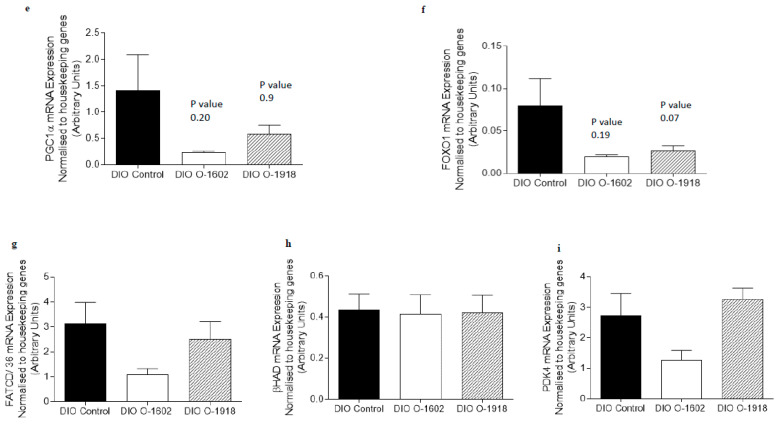
The abundance of mRNA expressed for G Protein-Coupled Receptor 18 and markers involved in adiponectin signalling, fatty acid metabolism and oxidative capacity in red gastrocnemius skeletal muscle obtained from rats fed a high fat diet for 9 weeks to induce obesity. The diet induced obese (DIO) control rats, the DIO O-1602 rats and the DIO O-1918 rats were treated via intraperitoneal injection for a further 6 weeks. mRNA expression was normalised to the average of housekeeping genes cyclophilin and βActin and grouped data is reported as mean (arbitrary units) ± SEM. Figure 1a The red gastrocnemius treatment groups compared to the white gastrocnemius group (* significance *p* < 0.05). Figure 1b–i The DIO control group is compared to either the DIO O-1602 group or the DIO O-1918 group. (**a**) G Protein-Coupled Receptor 18 (includes both DIO red and white gastrocnemius); (**b**) Adiponectin Receptor 1 (AdipoR1); (**c**) Adaptor protein containing pleckstrin homology domain, phosphotyrosine binding domain and leucine zipper motif 1 (APPL1); (**d**) Adaptor protein containing pleckstrin homology domain, phosphotyrosine binding domain and leucine zipper motif 2 (APPL2); (**e**) Peroxisome proliferator-activated receptor gamma co-activator 1 alpha (PGC1α); (**f**) Forkhead box protein 01 (FOXO1); (**g**) Fatty Acid Translocase/Cluster of Differentiation 36 (FATCD/36); (**h**) beta-hydroxyacyl-CoA dehydrogenase (βHAD); (**i**) Pyruvate Dehydrogenase Kinase 4 (PDK4).

GPR18 mRNA was identified in both red and white gastrocnemius skeletal muscle obtained from DIO rats, both in the presence and the absence of atypical cannabinoid compounds O-1602 or O-1918 (Figure 1a). The abundance of GPR18 expression was increased in the red gastrocnemius skeletal muscle when compared with the white gastrocnemius skeletal muscle (*p* < 0.05); however, treatment with atypical cannabinoids did not significantly alter the expression of this receptor.

### 2.2. Atypical Cannabinoids Effect on mRNA Expression of Genes Involved in Skeletal Muscle Metabolism in Red and White Gastrocnemius in DIO

**Figure 2:**
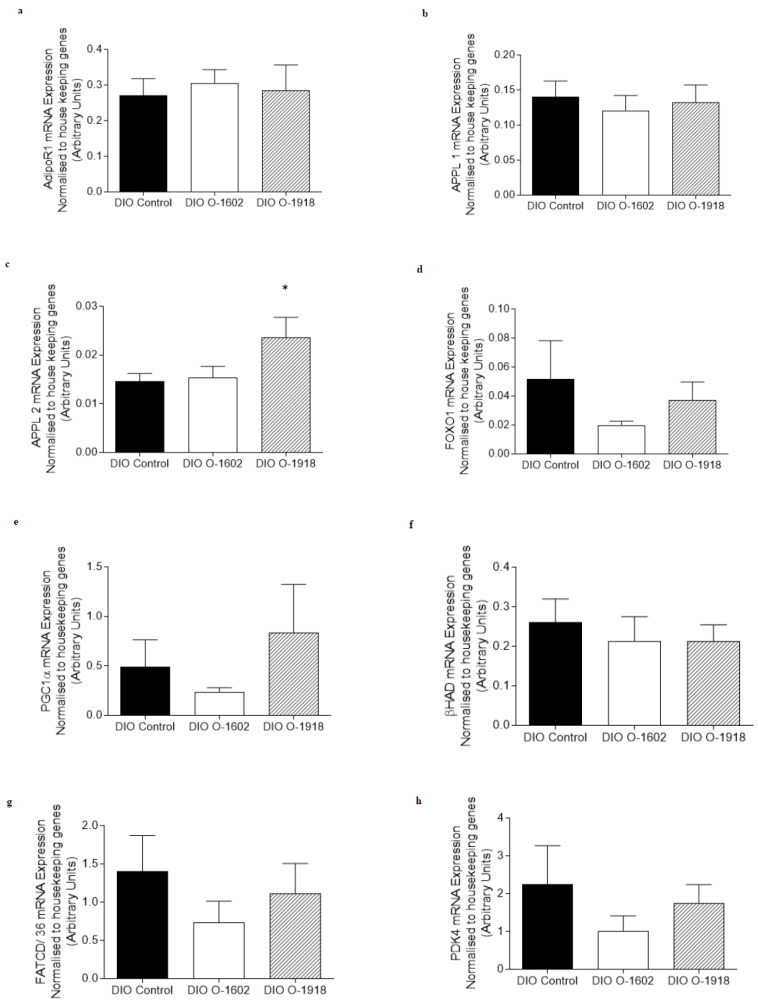
The abundance of mRNA expressed for markers involved in adiponectin signalling, fatty acid metabolism and oxidative capacity in white gastrocnemius skeletal muscle obtained from rats fed a high fat diet for 9 weeks to induce obesity. The DIO control rats, DIO O-1602 rats and the DIO O-1918 rats were treated via intraperitoneal injection for a further 6 weeks. mRNA expression was normalised to the average of housekeeping genes cyclophilin and βActin and grouped data is reported as mean (arbitrary units) ± SEM. The DIO control group is compared to either the DIO O-1602 group (* significance *p* < 0.05) or the DIO O-1918 group (* significance *p* < 0.05). (**a**) Adiponectin Receptor 1 (AdipoR1); (**b**) Adaptor protein containing pleckstrin homology domain, phosphotyrosine binding domain and leucine zipper motif 1 (APPL1); (**c**) Adaptor protein containing pleckstrin homology domain, phosphotyrosine binding domain and leucine zipper motif 2 (APPL2); (**d**) Forkhead box protein 01 (FOXO1); (**e**) Peroxisome proliferator-activated receptor gamma co-activator 1 alpha (PGC1α); (**f**) beta-hydroxyacyl-CoA dehydrogenase (βHAD); (**g**) Fatty Acid Translocase/Cluster of Differentiation 36 (FATCD/36); (**h**) Pyruvate Dehydrogenase Kinase 4 (PDK4).

The mRNA expression of genes involved in adiponectin, fatty acid metabolism and oxidative capacity signaling pathways were not altered by treatment with O-1602 in red or white gastrocnemius skeletal muscle obtained from DIO rats (Figure 1b–i and Figure 2a–h) when compared with the DIO control group. APPL2 mRNA expression was increased (*p* < 0.05) in white gastrocnemius skeletal muscle (Figure 2c) following treatment with O-1918 compared to the DIO control group.

The mRNA expression of all other genes measured involved in adiponectin, fatty acid metabolism and oxidative capacity signaling pathways were not altered in either red or white gastrocnemius skeletal muscle obtained from O-1918-treated DIO rats when compared with DIO control rats (Figure 1 and Figure 2).

### 2.3. Atypical Cannabinoids Effect on mRNA Expression of Genes Involved in Skeletal Muscle Metabolism in C_2_C_12_ Myotubes

**Figure 3:**
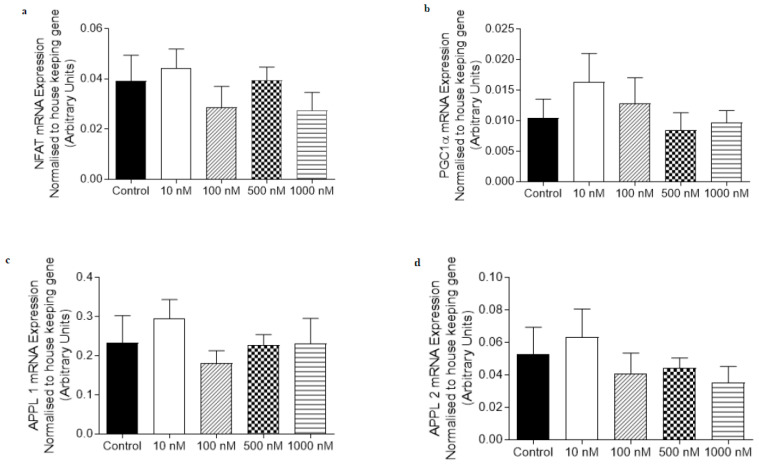
The abundance of mRNA expressed for markers involved in adiponectin signalling, fatty acid oxidation and oxidative capacity in C_2_C_12_ myotubes treated for 24 h with O-1602 (10–1000 nM). mRNA expression was normalised to housekeeping gene Hypoxanthine Phosphoribosyltransferase (HPRT1) and grouped data is reported as mean (arbitrary units) ± SEM. (**a**) Nuclear Factor of Activated T-Cells calcineurin dependent 1 (NFATc1); (**b**) Peroxisome proliferator-activated receptor gamma co activator 1-alpha (PGC1α); (**c**) Adaptor protein containing pleckstrin homology domain, phosphotyrosine binding domain and leucine zipper motif 1 (APPL1); (**d**) Adaptor protein containing pleckstrin homology domain, phosphotyrosine binding domain and leucine zipper motif 2 (APPL2).

**Figure 4:**
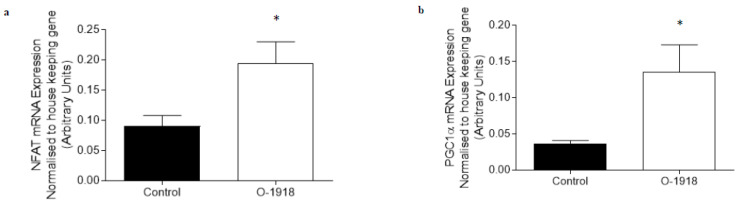

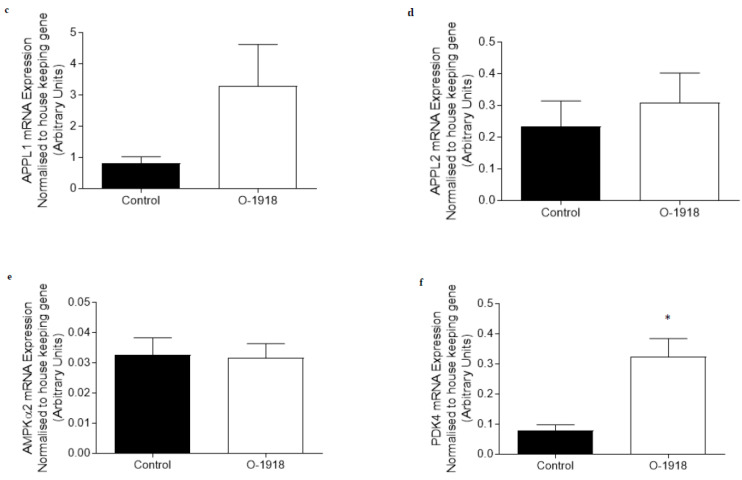
The abundance of mRNA expressed for markers involved in adiponectin signalling, fatty acid oxidation and oxidative capacity in C_2_C_12_ myotubes treated for 24 h with O-1918 (100 nM). mRNA expression was normalised to housekeeping gene Hypoxanthine Phosphoribosyltransferase (HPRT1) and grouped data is reported as mean (arbitrary units) ± SEM (* significance *p* < 0.05). (**a**) Nuclear Factor of Activated T-Cells calcineurin dependent 1 (NFATc1); (**b**) Peroxisome proliferator-activated receptor gamma co activator 1-alpha (PGC1α); (**c**) Adaptor protein containing pleckstrin homology domain, phosphotyrosine binding domain and leucine zipper motif 1 (APPL1); (**d**) Adaptor protein containing pleckstrin homology domain, phosphotyrosine binding domain and leucine zipper motif 2 (APPL2); (**e**) Adenosine Monophosphate Kinase alpha 2 (AMPKα2); (**f**) Pyruvate Dehydrogenase Kinase 4 (PDK4).

The mRNA expression of genes involved in adiponectin signaling (APPL1 and APPL2) and oxidative capacity (NFATc1 and PGC1α) were not altered by treatment with 10–1000 nM of O-1602 in C_2_C_12_ myotubes (Figure 3). Treatment with 100 nM of O-1918 on C_2_C_12_ myotubes caused an increase (*p* < 0.05) in the mRNA expression of NFATc1, PGC1α and PDK4 (Figure 4) when compared with the control group, while other markers including AMPKα2, APPL1 (*p* = 0.083) and APPL2 were not significantly altered when compared to the control group.

### 2.4. Effect of O-1918 on mRNA Expression of Oxidative Capacity and Adiponectin Signaling Genes in Human Primary Myotubes Obtained from Obese and Obese T2DM Individuals

**Figure 5:**
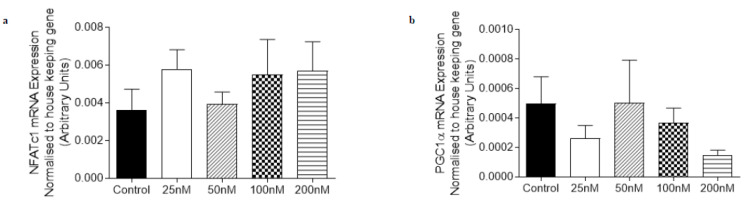

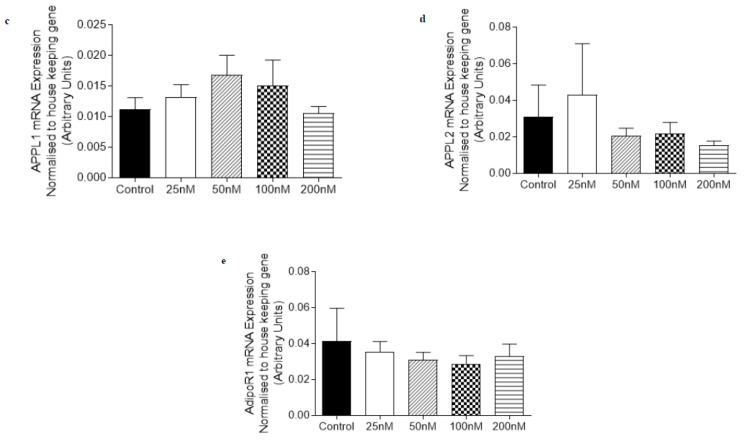
The abundance of mRNA expressed for markers involved in adiponectin signalling and oxidative capacity in human primary *rectus abdominus*-derived myotubes obtained from individuals that are obese treated for 24 h with O-1918 (25–200 nM). mRNA expression was normalised to housekeeping gene Cyclophilin and grouped data is reported as mean (arbitrary units) ± SEM. (**a**) Nuclear Factor of Activated T-Cells calcineurin dependent 1 (NFATc1); (**b**) Peroxisome proliferator-activated receptor gamma co activator 1-alpha (PGC1α); (**c**) Adaptor protein containing pleckstrin homology domain, phosphotyrosine binding domain and leucine zipper motif 1 (APPL1); (**d**) Adaptor protein containing pleckstrin homology domain, phosphotyrosine binding domain and leucine zipper motif 2 (APPL2); (**e**) Adiponectin Receptor 1 (AdipoR1).

**Figure 6:**
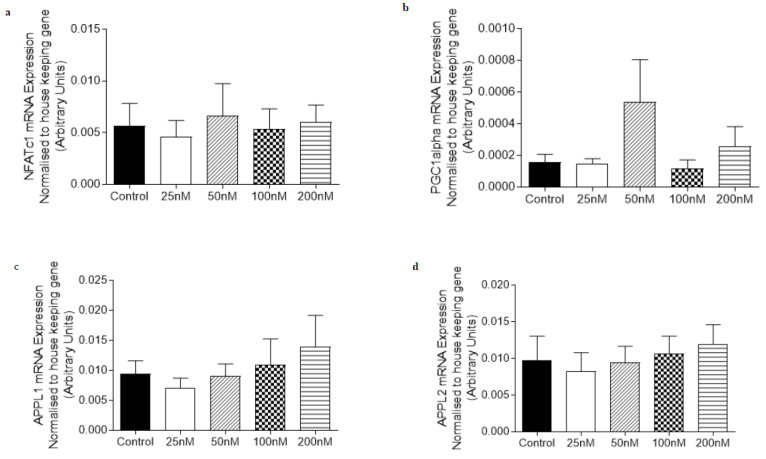

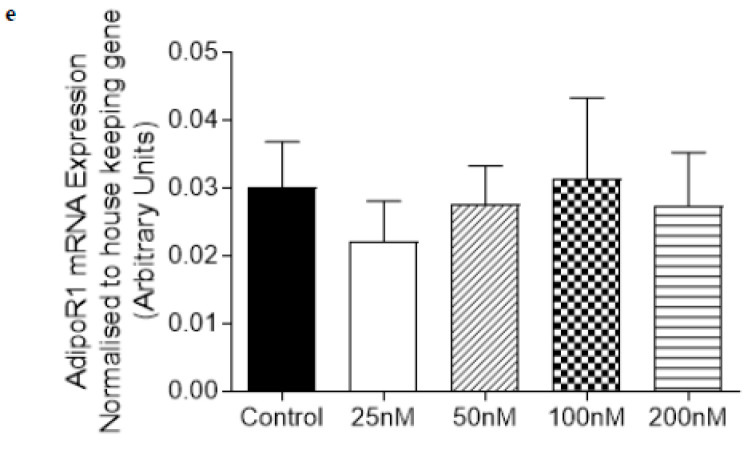
The abundance of mRNA expressed for markers involved in adiponectin signalling and oxidative capacity in human primary *rectus abdominus*-derived myotubes obtained from individuals that are obese and have type two diabetes mellitus treated for 24 h with O-1918 (25–200 nM). mRNA expression was normalised to housekeeping gene Cyclophilin and grouped data is reported as mean (arbitrary units) ± SEM. (**a**) Nuclear Factor of Activated T-Cells calcineurin dependent 1 (NFATc1); (**b**) Peroxisome proliferator-activated receptor gamma co activator 1-alpha (PGC1α); (**c**) Adaptor protein containing pleckstrin homology domain, phosphotyrosine binding domain and leucine zipper motif 1 (APPL1); (**d**) Adaptor protein containing pleckstrin homology domain, phosphotyrosine binding domain and leucine zipper motif 2 (APPL2); (**e**) Adiponectin Receptor 1 (AdipoR1).

Given our observations of O-1918 treatment on C_2_C_12_ myotubes and in the DIO rats, we then decided to determine the effect that O-1918 had on human primary myotubes derived from obese individuals and obese individuals with T2DM (Figure 5 and Figure 6). O-1918 did not have a significant effect on the mRNA expression of markers involved in oxidative capacity (NFAT or PGC1α) and adiponectin signaling (APPL1, APPL2 or AdipoR1) in either the obese or obese diabetic-derived myotubes.

## 3. Discussion

With the prevalence and incidence of obesity and related co-morbidities increasing world-wide [30], it is important to find different strategies to help address these health concerns. In addition to changes in dietary intake and physical activity, pharmacologically targeting the skeletal muscle to improve metabolic homeostasis [31] is a possible therapeutic strategy. We have recently shown that chronic administration of O-1602 or O-1918 has systemic effects in DIO rats [29]. Our previous study did not focus on the effects that these compounds had on the skeletal muscle, specifically, markers of fatty acid metabolism. Therefore, this study is the first to investigate atypical cannabinoid compounds O-1602 or O-1918 and their effect on skeletal muscle homeostasis in vitro in C_2_C_12_ myotubes and human primary myotubes, and in vivo in a DIO rat model.

Given that GPR55 is expressed in skeletal muscle obtained from rats [17] and appears to have a significant role in regulating insulin signalling [17], our study aimed to determine whether the other putative cannabinoid receptor, GPR18, was also expressed in skeletal muscle. The data included in this current study has shown for the first time that the putative cannabinoid receptor GPR18 is expressed in both red and white gastrocnemius skeletal muscle obtained from DIO rats. Our data suggests that GPR18 has a role in skeletal muscle metabolism, just like the traditional cannabinoid receptors CB_1_ and CB_2_, and the other putative cannabinoid receptor GPR55, all of which have previously been shown to be expressed in skeletal muscle [8,17] and all of which have a role in obesity [7,16,32].

Further, the results from this study showed that GPR18 mRNA expression was up-regulated in the red gastrocnemius when compared with the white gastrocnemius skeletal muscle, suggesting a variation in receptor expression between fibre types. Previous research shows that the CB1 receptor is down-regulated in soleus skeletal muscle obtained from obese Zucker rats when compared with lean Zucker rats [33]. In contrast, mice fed a high fat diet (HFD) for two months had up-regulated CB_1_ protein expression compared with standard chow-fed mice [34]. SR141716 treatment increased glucose uptake in the skeletal muscle of both standard chow- and high fat-fed mice [33]. CB_1_ receptors are mostly localised to the mitochondria in gastrocnemius and rectus abdominus skeletal muscle obtained from wild type mice, [35] although the effect that high fat feeding has on CB_1_ localisation within the mitochondria of skeletal muscle remains unclear. As GPR18 is up-regulated in red gastrocnemius in our study, this suggests that GPR18 may have a role in oxidative metabolism, however this finding is not reflective of cellular signalling and alterations that may occur as a result of receptor modulation, therefore further research into understanding the exact role of GPR18 in the skeletal muscle is required.

In addition to muscle type variation, systemic pharmacological treatment for six weeks with atypical cannabinoids O-1602 and O-1918 (compounds that have an affinity for the putative cannabinoid receptor GPR18 [11,13]), did not further alter the receptors’ mRNA expression in the DIO rats. Our results are similar to other cannabinoid research using AM251, an inverse agonist for the CB_1_ receptor, in which AM251 did not alter expression of the CB_1_ receptor in the skeletal muscle obtained from the abdominal wall of Wistar rats following two weeks of treatment, albeit these rats were fed a standard chow diet (SCD) and were not DIO [36]. In contrast, however, the same study did show an up-regulation of the other cannabinoid receptor CB_2_ with administration of the AM251 compound in SCD-fed Wistar rats [36]. In cardiac tissue obtained from streptozotocin (STZ)-induced male Wistar diabetic rats, a different atypical cannabinoid compound, abnormal cannabidiol (100 μg/kg), the analogue of O-1602, enhanced GPR18 protein expression (% control) following two weeks of treatment [28].

This study also aimed to determine whether the atypical cannabinoids O-1602 and O-1918 had an effect on skeletal muscle homeostasis in both C_2_C_12_ myotubes and human primary myotubes derived from obese or obese T2DM individuals (O-1918 only), as well as gastrocnemius skeletal muscle obtained from DIO rats. We have previously reported that rats fed a HFD for nine weeks have significantly greater body weight and body fat percentage when compared with rats fed a standard chow diet [37]. We have also reported that circulating concentrations of adiponectin were not altered by treatment with either O-1602 or O-1918 for six weeks in this DIO model [29]. Treatment for six weeks with O-1602, a biased agonist for GPR18 and an agonist for GPR55 [10,13] in DIO rats, caused no alterations in mRNA expression of markers involved in adiponectin signalling (AdipoR1, APPL1 and APPL2), fatty acid oxidation (FOXO1, βHAD, FATCD/36, PDK4) or oxidative capacity (PGC1α) in either the red or white gastrocnemius skeletal muscle. We have previously shown in this DIO model that O-1602 reduces total body fat and epididymal fat pad weight [29]. While we have demonstrated reduction in body fat in the DIO O-1602 rats as previously described [29], we did not observe any changes in the mRNA expression of any of the markers/pathways analysed in the skeletal muscle of these rats. In addition to the findings that O-1602 had on skeletal muscle in DIO, we also showed that O-1602 did not cause any alteration to markers of oxidative capacity (NFATc1 and PGC1α), or the positive regulator of adiponectin signalling APPL1 in C_2_C_12_ myotubes. Our study suggests that O-1602 does not appear to be effective in altering the skeletal muscle metabolism of these markers in the presence or absence of obesity.

Treatment with O-1918 in the red gastrocnemius did not alter markers of adiponectin signalling (AdipoR1, APPL1 and APPL2), markers of fatty acid metabolism (PDK4, FOXO1, βHAD, FATCD/36) or oxidative capacity (PGC1α). While in the glycolytic white gastrocnemius skeletal muscle, O-1918 up-regulated the mRNA expression of the negative regulator of adiponectin signalling, APPL2 [38]. However, in the white gastrocnemius skeletal muscle, O-1918 did not have any effect on other markers of adiponectin signalling (AdipoR1 or APPL1), fatty acid metabolism (FOXO1, βHAD, FAT/CD36 or PDK4) or oxidative capacity (PGC1α), while in the C_2_C_12_ myotubes, PGC1α, NFATc1, PDK4 and a trend for APPL1 were increased compared to control, which differed from the rat whole muscle tissue. The pharmacology for the atypical cannabinoid O-1918 is complex, as this compound acts as a putative antagonist for GPR55 and GPR18 [11], or as a biased agonist for GPR18 [13]. The fact that O-1918 enhances calcium mobilisation as a result of biased agonism at GPR18 [13] could help explain the up-regulation of mRNA for PGC1α [22] and NFAT, [23] and the trend for APPL 1 to be up-regulated [24] observed in our study. It has previously been reported that over expression of PGC1α in C_2_C_12_ myotubes resulted in activation of PDK4 mRNA and protein expression [25], and that over expression of PGC1α in C_2_C_12_ myotubes decreased the rate of glucose oxidation [25]. Therefore, the up-regulation of PGC1α mRNA expression observed in the C_2_C_12_ myotubes may also help to explain the up-regulation of PDK4 mRNA expression observed in our study.

## 4. Materials and Methods

### 4.1. Cell Culture

#### 4.1.1. C_2_C_12_ Myotubes

C2C12 cells were a kind gift from Professor David Cameron-Smith (Deakin University, Melbourne, Australia). Mouse-derived C_2_C_12_ myoblasts were cultured in Dulbecco’s modified eagle high glucose growth medium (D-MEM) supplemented with 10% foetal bovine serum (*v*/*v*), 1% penicillin streptomycin (*v*/*v*), 0.5% amphotericin B (*v*/*v*) and incubated at 37°C, 5% CO_2_ in a humidity controlled environment as previously described [39]. C_2_C_12_ myoblasts were seeded into 6 well plates and differentiated into myotubes (within ~72 h) via supplementation with 2% horse serum (*v*/*v*), 1% penicillin streptomycin (*v*/*v*), 0.5% amphotericin B (*v*/*v*) [39]. C_2_C_12_ cells were serum starved in 0.1% BSA (*w*/*v*) and a D-MEM solution for six hours prior to treatment, then treated for 24 h with vehicle (0.1% ethanol; Sigma Aldrich, St Louise, MO, USA) (*n* = 8–9) or O-1602 (10 nM–1000 nM; Cayman Chemical, Ann Arbor, Michigan, USA) (*n* = 9) or O-1918 (100nM; Cayman Chemical, Ann Arbor, Michigan, USA) (*n* = 8) and all treatments were dissolved in 0.1% ethanol and suspended in a 0.1% BSA and a D-MEM solution. Following treatment, cells were washed with ice cold PBS; then lysed, on ice with TRIzol Reagent^®^ (Invitrogen, Victoria, Australia) and stored at −80 °C for subsequent RNA extraction.

#### 4.1.2. Human Primary Rectus Abdominus Myotubes

The approval for the collection of rectus abdominus skeletal muscle samples from obese individuals and obese individuals with T2DM was approved by the ethics committees at both Victoria University (St Albans, Victoria, Australia) and The Avenue Hospital (St Kilda, Victoria, Australia) approval number HRETH 08/158 (07/10/2008) and Trial 0100 (22/06/2008), respectively. A portion of rectus abdominus skeletal muscle was obtained during abdominal surgery to establish human primary myotube culture. Donor characteristics were obtained and are included in Table 1.

Human primary myotubes were established as previously described [40], once myoblasts reached passage 4 they were then differentiated into myotubes and incubated for 2 h in serum-free 0.1% BSA (*w*/*v*) alpha minimum essential media (αMEM). Following this, myotubes were treated for 24 h with αMEM, 0.1% BSA and either a dose range of 25–200 nM O-1918 or vehicle control (0.1% ethanol). Following treatment, cells were washed with ice cold PBS; then lysed, on ice with TRIzol Reagent^®^ (Invitrogen, Carlsbad, California, USA) and stored at −80°C for subsequent RNA extraction.

### 4.2. Animal Care and High Fat Feeding

The approval of this study was obtained from the Animal Ethics Committee at the Howard Florey Institute (Parkville, Melbourne, Australia) (AEC 11-036). Seven week old male Sprague Dawley rats were purchased from the Animal Resource Centre (Canning Vale, Western Australia, Australia), then acclimatised to their new environment for at least seven days. Rats were singly housed for the duration of this study and fed a high fat diet HFD (21% fat diet by weight) [37,41] purchased from Specialty Feeds (Glen Forrest, Western Australia, Australia) and fed this diet for a total period of fifteen weeks. The first nine weeks of high fat feeding prior to treatment was to induce DIO [37].

#### O-1602 or O-1918 Pharmacological Intervention in DIO rats

The DIO rats continued the HFD for a subsequent six weeks following allocation into treatment groups, as previously described [29]. The treatment groups included; DIO Control (*n* = 11), DIO O-1602 (*n* = 6) and DIO O-1918 (*n* = 9). During the six weeks of pharmacological intervention, rats were administered a daily intraperitoneal (ip.) injection of either; a 0.75% Tween-80 saline solution (DIO Control), 5 mg/kg O-1602 (DIO O-1602) or 1 mg/kg O-1918 (DIO O-1918) dissolved in a 0.75% Tween-80 saline solution. O-1602 and O-1918 were sourced from Tocris Bioscience (Bristol, UK). The dose of O-1602 was selected due to the compound’s ability to reduce scores of colitis [42], while the dose of O-1918 was able to inhibit the hypotensive effects of abnormal cannabidiol [43].

Following the six week pharmacological treatment period, the rats were deeply anaesthetised using 3% isoflurane (Abbott, Botany, NSW, Australia), then red and white gastrocnemius skeletal muscles were surgically removed and a portion snap frozen in liquid nitrogen for subsequent analysis. Rats were then administered a lethal injection of 100 mg/kg sodium pentobarbitone (Virobac, Peakhurst, Australia) and euthanised via cardiac puncture.

### 4.3. RNA Extraction and cDNA Synthesis

Approximately 25–35 mg of red or white gastrocnemius skeletal muscle obtained from the pharmacologically treated DIO rats were utilised to extract RNA, as previously described [8,39]. RNA extracted from the rat tissue samples were treated with RQ1 RNAse-free DNAse kit (Promega Corporation, Madison, Wisconsin, USA) in accordance with manufacturer’s instructions. A total of 0.5 µg of RNA obtained from rat or cell culture was reverse transcribed into cDNA using the iScript^TM^ cDNA synthesis kit (BioRad Laboratories, Hercules, California, USA) in accordance with manufacturer’s instructions. In addition, 10 ng of cDNA was utilised for GPR18 expression and 2.5 ng of cDNA was utilised for analysis of all other genes involved in adiponectin signalling, fatty acid metabolism and oxidative capacity. The cDNA was stored at −20 °C.

Oligonucleotide primers were developed for selected genes using Oligoperfect Suite and then purchased from Geneworks Pty Ltd. (Adelaide, Australia). A BLAST search confirmed homologous binding for the target mRNA sequences. The forward and reverse oligonucleotide primer sequences for the genes of interest are detailed in Table 2.

### 4.4. ‘Real Time’ Polymerase Chain Reaction (PCR)

To quantify mRNA expression of the genes of interest, ‘Real Time’ PCR, SYBR Green method [44] was utilised. SYBR^TM^ Green (BioRad Laboratories, Hercules, California, USA) and the BioRad MY iQ^®^ Real-Time PCR detection system were used. Forward and reverse oligonucleotide primer sequences for mouse, rat and human are included in Table 2. The samples were run for 40 or 50 cycles at 95 °C for 15 s and 60 °C for 60 s. Changes in mRNA expression were normalised to the average of housekeeping gene(s), cyclophilin and β actin in rat muscle, HPRT1 in C_2_C_12_ myotubes and cyclophilin in human primary myotubes and quantified using the validated 2^−ΔΔct^ method. The data was reported in arbitrary units as previously described [45].

### 4.5. Statistical Analysis

Graph Pad Prism Software 8.1.2 was used to generate figures and perform statistical analysis, all grouped data is reported as mean ± SEM. The normality of the data was assessed using the Shapiro–Wilk Test. Normally distributed data were statistically analysed using an independent two tailed *t*-test and not normally distributed data were analysed using a Mann–Whitney two tailed test to determine the effect of treatment compared to control or each treatment group when comparing red gastrocnemius to white gastrocnemius skeletal muscle. A one way ANOVA and Tukey’s Multiple Comparisons Test was utilised to compare the treatment groups and control groups for the red or white gastrocnemius skeletal muscle GPR18 expression data as well as the human primary and C_2_C_12_ myotubes. Statistical significance for all data sets was accepted at *p* < 0.05.

## 5. Conclusions

Collectively, this is the first study to investigate the effect of two atypical cannabinoid compounds, O-1602 or O-1918, on the skeletal muscle homeostasis in a DIO model, metabolically stable C_2_C_12_ myotubes and human primary myotubes obtained from individuals that were obese or obese and had T2DM (O-1918 only). The results from this study suggest that O-1602 does not have an effect on the mRNA expression of a number of signalling pathways in the skeletal muscle under normal physiological conditions or in obesity. While O-1918 appears to have variable effects on skeletal muscle metabolism, the up-regulation of PDK4 in the in vitro model suggests a potential benefit for fatty acid metabolism. Oxidative metabolism markers PGC1α and NFATc1 were also up-regulated in the in vitro model; however, no alterations were observed in the in vivo DIO model. APPL2, the negative regulator of adiponectin signalling, was up-regulated in glycolytic white gastrocnemius skeletal muscle in the in vivo DIO model. The variation between findings could be due to a number of reasons such as species differences in receptor structure, function, activity and expression, and in vivo systemic changes affecting skeletal muscle function versus targeted in vitro administration of treatments. Therefore, in conclusion, O-1602 does not appear to be a suitable skeletal muscle target for obesity at the dosage and duration selected in the current study, while localised O-1918 treatment may be beneficial in targeting pathways of oxidative capacity and fatty acid metabolism in myotubes, albeit only in C_2_C_12_ myotubes. Further, based on our findings, future research focusing on mitochondrial count and membrane potentials, myogenesis markers (such as myostatin I, IIa and IIb, glycogen synthsis (GSK3b) and other markers of oxidative capacity (such as NRF2 and SOD1) may be of benefit.

Given that GPR18 is expressed in the skeletal muscle, selectively targeting this receptor in a tissue-specific manner, and understanding how its function may be altered in obesity through different associated mechanisms and the implications of different dietary interventions on receptor expression, would be beneficial in the search for targeted therapies for obesity and related co-morbidities.

## Figures and Tables

**Table 1:** Characteristics for donors of rectus abdominus skeletal muscle.

Characteristic	Group	
	Obese (*n* = 8)	Obese Diabetic (*n* = 8)
**Sex**	Female *n* = 5 Male *n* = 3	Female *n* = 5 Male *n* = 3
**Age (years)**	45.9 ± 4.9	48.6 ± 3.5
**Weight (kg)**	106.9 ± 6.8	114.2 ± 7.3
**Height (m)**	1.6 ± 0.0	1.7 ± 0.0
**BMI**	40.2 ± 1.8	39.5 ± 1.8
**Fasting Blood Glucose (mmol/L)**	5.3 ± 0.1	10.8 ± 1.5 *
**Plasma Insulin (µU/L)**	9.5 ± 1.4	15.6 ± 2.6
**Hba1c %**	5.5 ± 0.1	8.8 ± 0.8 *
**Cholesterol**	5.1 ± 0.5	4.9 ± 2.6
**Fasting Triglycerides**	1.4 ± 0.2	2.6 ± 0.6
**HDL-cholesterol**	1.4 ± 0.1	1.1 ± 0.1 *
**LDL-cholesterol**	3.0 ± 0.4	3.2 ± 0.5

Values are expressed as means ± SEM. * Indicates a significant difference between obese and obese Diabetic groups (*p* < 0.05).

**Table 2:** Forward and Reverse Oligonucleotide Primer Sequences for ‘Real Time’ Polymerase Chain Reaction.

Primer	Accession Number	Direction	Sequence
Rat Genes			
*Cyclophilin*	NM_017101.1	Forward (5′ 3′)	CTG ATG GCG AGC CCT TG
		Reverse (5′ 3′)	TCT GCT GTC TTT GGA ACT TTG TC
*β-Actin*	NM_031144	Forward (5′ 3′)	CTA AGG CCA ACC GTG AAA TGA
		Reverse (5′ 3′)	CCA GAG GCA TAC AGG GAC AAC
*GPR18*	NM_001079710.1	Forward (5′ 3′)	GTG GGG GTC TGG ATA ATG AC
		Reverse (5′ 3′)	CGC GTG AAG TTA AGC ACA TT
*AdipoR1*	NM_207587.1	Forward (5′ 3′)	TGA GGT ACC AGC CAG ATG TC
		Reverse (5′ 3′)	CGT GTC CGC TTC TCT GTT AC
*APPL1*	XM_008771023.1	Forward (5′ 3′)	TCA CTC CTT CCC CAT CTT TC
		Reverse (5′ 3′)	TAG AGA GAG GGC AGC CAA AT
*APPL2*	NM_001108741.1	Forward (5′ 3′)	TGC TCG GGC TAT TCA CAA
		Reverse (5′ 3′)	AAA CAG GCC CGT GAC ACT
*PGC1α*	NM_031347.1	Forward (5′ 3′)	ACC CAC AGG ATC AGA ACA AACC
		Reverse (5′ 3′)	GAC AAA TGC TCT TTG CTT TAT TGC
*FOXO1*	NM_001191846.2	Forward (5′ 3′)	CTC GGC GGG CTG GAA
		Reverse (5′ 3′)	TCA TTC TGT ACT CGA ATA AAC TTG
*PDK4*	NM_053551.1	Forward (5′ 3′)	GGG ATC TCG CCT GGC ACT TT
		Reverse (5′ 3′)	CAC ACA TTC ACG AAG CAG CA
*βHAD*	AF095449.1	Forward (5′ 3′)	TCG TGA CCA GGC AAT TCG T
		Reverse (5′ 3′)	CCG ATG ACC GTC ACA TGC T
*FAT/CD 36*	NM_031561.2	Forward (5′ 3′)	GAC CAT CGG CGA TGA GAA A
		Reverse (5′ 3′)	CCA GGC CCA GGA GCT TTA TT
Mouse Genes			
*HPRT1*	NM_013556.2	Forward (5′ 3′)	GCAAACTTTGCTTTCCCTGG
		Reverse (5′ 3′)	ACTTCGAGAGGTCCTTTTCAC
*NFATc1*	NM_016791.3	Forward (5′ 3′)	TCCAAAGTCATTTTCGTGGA
		Reverse (5′ 3′)	GTTGCGGAAAGGTGGTATCT
*PGC1α*	NM_008904.1	Forward (5′ 3′)	CACCCACAGGATCAGAACAA
		Reverse (5′ 3′)	GGTCATCGTTTGTGGTCAGA
*APPL1*	NM_145221.2	Forward (5′ 3′)	ATCAGGCGGAAGAAGTGAGA
		Reverse (5′ 3′)	TTTCTGATGCCCTACGATCC
*APPL2*	NM_145220.2	Forward (5′ 3′)	CCAAAAGTATGGACGGCTTC
		Reverse (5′ 3′)	CTCAGCTTCCAGTTCCACCT
*AMPKα2*	NM_178143.1	Forward (5′ 3′)	GCCCAGATGAACGCTAAGAT
		Reverse (5′ 3′)	TGCATACAGCCTTCCTGAGA
*PDK4*	NM_013743.2	Forward (5′ 3′)	GAGAAGAGCCCAGAAGACCA
		Reverse (5′ 3′)	TCCACTGTGCAGGTGTCTTT
Human Genes			
*Cyclophilin*	NM 021130.3	Forward (5′ 3′)	CATCTGCACTGGCAAGACTGA
		Reverse (5′ 3′)	TTCATGCCTTCTTTCACTTTGC
*NFATc1*	NM_172390.1	Forward (5′ 3′)	CCT CTC CAA CAC CAA AGTCC
		Reverse (5′ 3′)	CGA TGT CCG TCT CTC CTT TC
*PGC1α*	NM_013261	Forward (5′ 3′)	CAAGCCAAACCAACAACTTTATCTCT
		Reverse (5′ 3′)	CACACTTAAGGTGCGTTCAATAGTC
*AdipoR1*	NM_015999	Forward (5′ 3′)	CGCCATGGAGAAGATGGAA
		Reverse (5′ 3′)	TCATATGGGATGACCCTCC
*APPL1*	NM_012096	Forward (5′ 3′)	TCACTCCTTCCCCATCTTTC
		Reverse (5′ 3′)	TAGAGAGAGGGCAGCCAAAT
*APPL2*	NM_018171	Forward (5′ 3′)	CACGCCCAATGGAAAATC
		Reverse (5′ 3′)	CGACTGCCTCAGGGTTGT

AdipoR1; Adiponectin Receptor 1, AMPKα2; 5′adenosine monophosphate-activated protein kinase α-2, APPL1; Adaptor protein, phosphotyrosine interacting with PH domain and leucine zipper 1, APPL2; Adaptor protein, phosphotyrosine interacting with PH domain and leucine zipper 2, βActin; beta actin, βHAD; beta-hydroxyacyl-CoA dehydrogenase, FAT/CD36; Fatty Acid Translocase/ Cluster of Differentiation 36, FOXO1; Forkhead box protein O1, GPR18; G Protein-Coupled Receptor 18, HPRT1; Hypoxanthine-Guanine Phosphoribosyltransferase, NFATc1; nuclear factor of activated T-cells c1, PDK4; Pyruvate Dehydrogenase Kinase 4, PGC1α; Peroxisome proliferator-activated receptor gamma co-activator 1 alpha.

## Abbreviations

GPR55	G Protein Coupled Receptor 55
GPR18	G Protein Coupled Receptor 18
DIO	Diet-Induced Obese
APPL2	Adaptor protein, phosphotyrosine interacting with PH domain and leucine zipper 2
PGC1α	Peroxisome proliferator-activated receptor gamma co-activator 1 alpha
NFATc1	Nuclear factor of activated T-cells c1
PDK4	Pyruvate Dehydrogenase Kinase 4
T2DM	Type Two Diabetes Mellitus
CB_1_	Cannabinoid Receptor 1
CB_2_	Cannabinoid Receptor 2
GPCR	G Protein-Coupled Receptor
LPI	Lysophosphatidylinositol
MIN6	Mouse insulinoma
APPL1	AdipoR1; Adiponectin Receptor 1
CBD	Cannabidiol
Abn-CBD	Abnormal Cannabidiol
SCD	Standard Chow Diet
STZ	Streptozotocin
AdipoR1	Adiponectin Receptor 1
FOXO1	Forkhead box protein O1
βHAD	Beta-hydroxyacyl-CoA dehydrogenase
FATCD/36	Fatty Acid Translocase/Cluster of Differentiation 36
D-MEM	Dulbecco’s modified eagle high glucose growth medium
α-MEM	Alpha minimum essential media
HFD	High Fat Diet
RNA	Ribonucleic Acid
cDNA	Complementary deoxyribonucleic acid
